# Global Perspectives of Oral Health Policies and Oral Healthcare Schemes for Older Adult Populations

**DOI:** 10.3389/froh.2021.703526

**Published:** 2021-08-16

**Authors:** Chloe Meng Jiang, Chun Hung Chu, Duangporn Duangthip, Ronald L. Ettinger, Fernando Neves Hugo, Matana Kettratad-Pruksapong, Jian Liu, Leonardo Marchini, Gerry McKenna, Takahiro Ono, Wensheng Rong, Martin Schimmel, Naseem Shah, Linda Slack-Smith, Stella X. Yang, Edward C. M. Lo

**Affiliations:** ^1^Faculty of Dentistry, The University of Hong Kong, Hong Kong, China; ^2^The University of Iowa College of Dentistry and Dental Clinics, Iowa City, IA, United States; ^3^Department of Preventive and Social Dentistry, Federal University of Rio Grande do Sul, Porto Alegre, Brazil; ^4^Faculty of Dentistry, Thammasat University, Bangkok, Thailand; ^5^Peking University School & Hospital of Stomatology, Beijing, China; ^6^Centre for Public Health, Queen's University Belfast, Belfast, United Kingdom; ^7^Division of Comprehensive Prosthodontics, Niigata University Faculty of Dentistry & Graduate School of Medical and Dental Sciences, Niigata, Japan; ^8^Department of Reconstructive Dentistry and Gerodontology, School of Dental Medicine, University of Bern, Bern, Switzerland; ^9^Division of Gerodontology and Removable Prosthodontics, University Clinics of Dental Medicine, University of Geneva, Geneva, Switzerland; ^10^Centre for Dental Education and Research, All India Institute of Medical Sciences, New Delhi, India; ^11^School of Population and Global Health, The University of Western Australia, Perth, WA, Australia

**Keywords:** international health, healthcare policy, dental care, older adults, aging

## Abstract

The aim of this study was to present a concise summary of the oral health policies and oral healthcare schemes for older adult populations in a number of selected countries around the world. In this paper, the current and planned national/regional oral health policies and oral healthcare schemes of nine countries (Australia, Brazil, China including Hong Kong, India, Japan, Switzerland, Thailand, the United Kingdom, and the United States) are reported. Barriers and challenges in oral health promotion in terms of devising oral health policies, implementing oral health schemes, and educating the future dental workforce are discussed. In response to the aging of population, individual countries have initiated or reformed their healthcare systems and developed innovative approaches to deliver oral health services for older adults. There is a global shortage of dentists trained in geriatric dentistry. In many countries, geriatric dentistry is not formally recognized as a specialty. Education and training in geriatric dentistry is needed to produce responsive and competent dental professionals to serve the increasing number of older adults. It is expected that oral health policies and oral healthcare services will be changing and reforming in the coming decades to tackle the enduring oral health challenges of aging societies worldwide.

## Introduction

The number and percentage of older adults in most countries around the world are rapidly increasing. The United Nations estimates that there were 703 million older adults (aged 65 years or above) globally in 2019 and this number will double to 1.5 billion by 2050 [1]. The estimated proportion of older adults in 2019 was 9% globally and it is expected to increase to 16% by 2050. In the coming decades, the rate of population aging will be the highest in Northern Africa and Western Asia while the lowest will be in Europe and North America where the population is already significantly older than in other parts of the world [1]. Despite the variation in different geographical regions, aging of the population is a global phenomenon that cannot be neglected.

Oral health, as an essential component of health, is a fundamental human right. It contributes to physical, mental, and social well-being of a person and has a significant impact on the quality of life [2]. Multifaceted and complex links between oral health and general health have been identified [3]. Systemic diseases can influence oral health, directly *via* their pathological processes and/or indirectly *via* disease-related behavioral changes, while oral diseases such as periodontitis are associated with systemic diseases such as endocarditis [4] and diabetes mellitus [5]. Prevention of oral diseases and promotion of oral health contribute to better general health for older adults, which can improve their quality of life.

The burden of oral diseases is substantial globally [6]. Dental caries and periodontitis are prevalent diseases among adults worldwide. It was estimated that in mid-2010s 35% of the global population were affected by untreated caries, while 11% of adults had severe periodontal diseases [7, 8]. Extensive tooth loss, which increases exponentially with age, is another heavy burden for an aging society [9]. According to the 2019 Global Burden of Diseases, Injuries, and Risk Factors Study (GBD), oral disorders affect more than 280 million older adults aged 70 years or more, and it has been one of the leading causes of global disability-adjusted life-years (DALYs) [10]. In a recent review, the estimated global prevalence and incidence of oral conditions (i.e., untreated dental caries, periodontitis, edentulism, and other oral disorders) of older adults were 57 and 77%, respectively [6].

Notwithstanding regional differences, most countries are moving forward to achieve a healthy aging society at their own pace by adopting tailor-made strategies. There has been constant improvement in the performance of healthcare systems globally over the last three decades, but important inequalities persist and the gap between high- and low-income countries has not been reduced [11]. In this international collaborative project, we aimed to present a concise summary of the national and regional oral health policies, and oral healthcare schemes for older adults in selected countries around the world, so that the readers can have a general view of the global situation.

## Methods

Authors from nine selected countries (Australia, Brazil, China including Hong Kong, India, Japan, Switzerland, Thailand, the United Kingdom, and the United States of America) were invited to report on the oral health policies and oral healthcare schemes for older adults in their corresponding country/region. The authors followed an agreed outline to present the situation of their own country/region. Firstly, the most recent available key epidemiological information regarding the oral health conditions (dental caries, periodontal status, and tooth loss) of the older adults was summarized. Secondly, the current national/regional oral health policies and oral healthcare schemes/services for older adults, if present, were described. In addition, local situations regarding dental professionals specialized in geriatric dentistry and advanced training in geriatric dentistry were reviewed. Lastly, obstacles and barriers for developing appropriate dental care services for the older adults in the country/region, as well as possible future plans, were briefly discussed.

## Results

### Australia

Australia, like many nations, has an aging population and groups of older adults that are highly disadvantaged in terms of oral health and access to dental care [12]. The Australian government identifies four groups in particular: (1) socially disadvantaged or low income; (2) Indigenous Australians; (3) rural and remote dwellers, and (4) groups of older adults with special needs [13]. The limited data available on oral health in older adults comes from large research projects and national oral health surveys. In the Concord Health and Aging in Men Project, older Australian men had a positive view of their oral health and most of those identified clinically as needing treatment did not perceive the need for treatment themselves [14]. The 2017–18 national oral health survey identified that the proportion of Australians over 75 years with complete tooth loss as 20.5% [15]. Other available key measures from national surveys are shown in Table 1.

**Table 1:** Summary of oral health status of older adult populations in the selected countries/regions.

**Country/Region**	**Year of survey**	**Caries status**	**Periodontal status—% with pockets**	**Edentulous rate**	**Mean number of missing teeth**	**References**
Australia	2017–2019	17.8% of older adults (over 75 years) had untreated caries	–	20.5%	4.8	[15–17]
	2004–2006	DMFT: 23.7 (over 65 years old)	53.4% (over 65 years) 60.8 % (over 75 years)	–	–	[18, 19]
Brazil	2010	DMFT: 27.5	CPI = 4: 3.3%	53.7%	25.3	[20]
China	2015–2016	DMFT > 0: 98% DMFT: 13.3	14.7% had 6+mm pockets	4.5%	9.5	[21]
Hong Kong	2011	47.8% (65–74 years old) had untreated caries DMFT: 16.2	59.2% dentate persons had 4+mm pockets	5.6%	–	[22]
India	2014	50.1% DT: 3.8	79.4%	–	–	[23]
	2007	–	–	65–74 years old: 18.5%	–	[24]
Japan	2016	DMFT: 65–69 years old: 18.8 70–74 years old: 19.6 75–79 years old: 21.2 80–84 years old: 23.3 85+ years old: 24.8	65–74 years old: 57.5% 75+ years old: 50.6%	65–69 years old: 2.4% 70–74 years old: 6.3% 75–79 years old: 7.5% 80–84 years old: 16.1% 85+ years old: 27.2%	–	[25]
Switzerland	2012	–	–	65–74 years old: 6.5% 75–84 years old: 8.6% 85+ years old: 15.1%	65–74 years old: 7 75–84 years old: 9 85+ years old: 12	[26]
	2011	DMFT: 26.8	–	19%	16.5	[27]
	2012	–	95%	52%	–	[28]
Thailand	2017	52.6% of older adults aged 60–74 years had untreated caries DMFT: 15.9 DT = 1.8	24.1% (4–5 mm PD) 12.2% (6 mm PD)	8.7%	13.1	[29]
		43.5% of older adults aged 80–85 years had untreated caries DMFT: 24.0 DT = 1.8	10.6% (4–5 mm PD) 5.9% (6 mm PD)	31%	21.9	[29]
UK	2009	DT 65–74 years old: 0.9 75–84 years old: 1.0 85 + years old: 0.7	Pocket and LoA of 4 mm or more 65–74 years old: 77% 75–84 years old: 82% 85 + years old: 76%	65–74 years old: 15% 75–84 years old: 30% 85 + year old: 47%	–	[30]
US	2015–2018	–	–	12.9%	–	[31]
	2011–2016	16% of the older adults had untreated caries DMFT: 16.7	–	–	–	[32]
	2009–2012	–	62.3% with at least one site ≥5 mm	–	–	[33]

Australian policies regarding oral health of older adults have increasingly focused on this intersectoral, interdisciplinary issue with greater input from areas such as public health and primary health care. In Australia, dental care is not generally included in the universal healthcare system Medicare [34]. Australia has a national oral health plan [35], but has limited number of dental professionals trained to care for persons with special needs and does not recognize geriatric dentistry as a specialty. The federal government funds broad dental services periodically but most government dental care is delivered by states with varied policies. Generally, older adults with low income (eligible for a government pension) are eligible for public dental care but often with a co-payment [34]. Findings from a recent review showed that training in geriatric dentistry in Australian dental schools has been limited and potentially behind many parts of the world [36]. Barriers in engagement regarding dental care in residential aged care have been identified and summarized as manager involvement, dental professional (dentist and oral health therapist) involvement, nursing care involvement, and family/carer involvement [37].

There have been limited large-scale programs addressing the oral health of older Australians, both in the community and in residential care, apart from subsidized public dental care. One review noted most workforce models for dental care in aged care have some benefits in terms of dental outcomes [38]. One approach which is considered very promising in Australia involves placing oral health therapists in residential aged care facilities to support dental hygiene and referral [39, 40].

### Brazil

The oral health of older adults in Brazil has been characterized by a high prevalence of edentulism. According to the 2010 Brazilian Oral Health Survey, the latest available survey from a nationally representative sample, 53.7% of the adults aged 65–74 years were edentulous [20]. This survey also revealed a strong social gradient, with older adults from the lower socioeconomic groups experiencing edentulism five times more frequently than older adults from the highest group [41]. The prevalence of root caries among older adults was 13.6% [42]. The 2019 GBD study revealed a prevalence of edentulism of 68.1% with 7.9 million Brazilians aged 70 or above experiencing edentulism, amounting to more than 211,000 years lived with disability. In the same study, the prevalence of untreated dental caries and periodontitis in older adults were 22.2 and 14.5%, respectively [10].

Brazil reformed its healthcare system in 1988, transitioning to universal health care with the development of the Sistema Único de Saúde (Unified Health System). Under this system, Brazilians are entitled to free health care at government funded facilities. Incentives for the implementation of oral health teams within primary care started in 2000 and a national oral health policy was implemented in 2004. Older adults are prioritized as a group, with preferential access to oral health care. Primary health care is the main source of oral health care for the older adult population, and services include oral health promotion and prevention, dental clinical care, and oral rehabilitation with partial and complete dentures provided by oral health teams. The oral health network also comprises specialized dental care centers, and older adults are referred, if needed, by the oral health teams to receive specialized care in endodontics, periodontics, oral surgery, and oral medicine [43]. Geriatric dentistry has been recognized as a specialty in Brazil since 2001, and a survey in 2007 revealed that the country had 18 certificate programs [44]. Since then, the specialty has not gained momentum, as there are only 275 specialists working across the country, a number that is certainly much smaller than what is required.

The Brazilian oral health policy expanded access to oral health care for older adults, with more than 27,000 oral health teams in primary care and more than 1,000 specialized dental care centers providing oral health care free of charge [43]. In addition, the number of older adults who had never visited a dentist reduced sharply. However, the policy still has many challenges, particularly in relation to access. Population coverage for oral health services has been stable at ~40% of the population in the last 5 years. Specifically, partial and complete tooth loss among older adults is still a major oral diseases burden, while an inefficient bureaucracy is another challenge faced by local health authorities that are willing to provide the much needed oral rehabilitation services.

### China

In mainland China, there were 264 million older adults aged 60 and above in 2020, which accounted for 18.7% of the total population [45]. The fourth national oral health survey conducted in 2015–2016 revealed a high prevalence of dental caries among the people aged 65–74 years, while 98.0% of the older adults had caries experience and 87.2% of the caries remained untreated [21]. For older adults aged 65–74 years old, the mean decayed, missing, filled teeth (DMFT) score was 13.3 ± 9.3 and the filling rate was 12.8%. Only 9.3% of the older adults had no measurable periodontal problems, while 15% had deep pockets (periodontal probing depth ≥ 6 mm). Over 30% of the older adults had lost at least five teeth (excluding the third molars), 48% had missing teeth which were not replaced, and almost 5% were edentulous [46].

Dental health care in China is predominantly provided by hospital-based public health services at the provincial, county and rural area level [47]. The majority of the population including older adults are covered by three basic social medical insurance systems, the “Basic Medical Insurance of Urban Population” (BMIUP) for the urban residents, the “New Rural Cooperative Medical System” (NRCMS) in the rural areas and the “Government Medical Insurance” for government officials. In 2011, only 5.2% of the total population were not covered by basic social medical insurance [48]. Dental treatment costs, especially for prosthodontic treatment is not fully covered by the medical insurance systems [49]. In order to reduce the burden of oral diseases among low-income people, local governments, and charitable organizations have launched free denture projects for people over 60 years old. In 2009–2012, edentulous older adults with low-incomes in Beijing had benefited from this “free denture” program for four consecutive years [50].

Recently, geriatric dentistry has drawn attention as a new subject. Some dental schools started a new curriculum including geriatric dentistry. For example, students in the Peking University School of Stomatology are required to participate in community projects every year as part of their dental public health curriculum. They are trained to conduct oral examinations for older adults and to understand older adults' oral health beliefs, knowledge, and behaviors through focus group discussions. The students are also required to design oral health pamphlets and to deliver oral health educational talks to older adults.

Despite the large treatment needs, utilization of dental services is very limited among older adults in China. The main reasons for dental neglect include lack of oral health knowledge, economic problems, perceived lower priority for oral healthcare by individuals and limited dental care resources [47, 49]. To address the above problems, the General Office of the National Health Commission issued “The Healthy Oral Action Plan (2019–2025),” advocating older adults to pay attention to their own oral health which is closely related to their general health. This long-term program aims to: (1) enhance oral healthcare services for older adults with chronic diseases through organized projects on prevention and treatment of dental caries, periodontal diseases as well as oral mucosal diseases; and (2) improve the competency of general dental practitioners in community oral health care institutions. The content of oral health will be included in the existing chronic disease monitoring system from 2021 onwards. Information about oral diseases will be collected and investigated every 5 years, so that a national oral health-monitoring network will be gradually established.

### Hong Kong

The oral health status of older adults in Hong Kong is far from satisfactory. Dental caries, periodontal diseases and tooth loss are prevalent among the older adults [22] (Table 1). There is no specific oral healthcare policy for older adults in Hong Kong. The government's oral healthcare policy for the general population is to raise the awareness of oral health and to encourage good oral health behaviors through health promotion and education [51]. Notwithstanding this, two public funded dental care service programs have been implemented for the older population for around 10 years [52]. One is the Outreach Dental Care Program (ODCP) for older adults using long-term care (LTC) services in elderly residential homes and day care centers. The ODCP is fully funded by the government Department of Health but is administered mainly by non-governmental organizations (NGOs) which have established dental clinics. After obtaining financial support from the government, the NGO set up an outreach dental service team which usually consists of one dentist, one dental surgery assistant and a general helper. The outreach dental service team visits the LTC facilities to provide free dental services for older adults, which include on-site dental check-up, prevention, scaling, pain relief and emergency treatments. On-site oral care training and oral health education activities such as seminar/talks are provided for older adults, their family members and caregivers. Funded by the government, further dental treatments are arranged and provided in the NGO's dental clinics to the ODCP participants who need tooth extractions, dental restorations, and dentures. The other program is the Community Care Fund Elderly Dental Assistance Program which is for older adults aged 60 years or above who are not receiving any government social welfare allowances but are covered by the subsidized community or home care services schemes, and for the recipients of the Old Age Living Allowance (a government means-tested scheme for older adults who are aged 65 years or older). The subsidized dental service, with a maximum amount set at HK$ 15,350 (US$ 1,970), can be provided by registered dentists working in private practice or NGO dental clinics. This service includes providing new dentures and any necessary denture-related services, such as radiographic examination, scaling, tooth extraction, and restoration.

The above two oral care service programs aim to provide primary prevention (e.g., topical fluoride application), secondary prevention (e.g., scaling), and tertiary prevention (e.g., denture) of oral diseases for selected older adults in Hong Kong. The shared funding, administration and provision mode of dental care services for the older adults makes good use of public funds and the private dental care delivery system in Hong Kong. With continued improvements in the scope of treatment, expansion of the eligible population and increases in subsidies for these programs, the number of older adults benefiting from this shared mode of dental service provision has been increasing over the years [53]. However, shortage of dental personnel, lack of expertise in geriatric dentistry, insufficient collaboration between dental and other primary healthcare workers, and inadequate oral health care training for healthcare providers are the main barriers to be tackled by the dental and healthcare professionals and policymakers.

### India

India is the world's second most populous country comprising 17% of the total world population. Of its 1.36 billion people, 116 million (9%) are above the age of 60 years, and this age group will likely increase to over 324 million by 2050 [1]. Three quarters of the population reside in rural areas and 90% work as casual laborers with no income security in old age. The older adults in India commonly suffer from multiple chronic diseases and disabilities, and a high proportion of the elderly widows live in poverty, social neglect, and economic dependence.

Under the National Health Mission, the National Oral Health Programme (NOHP) was launched in 2013. The nodal agency is the National Oral Health Cell at the Ministry of Health and Family Welfare. The state and district level oral health cell receives funding to set up one dental clinic with the required manpower, equipment, and consumables, in addition to the existing dental care facilities at the district level or below. The National Oral Health Cell at the central level is responsible for planning, monitoring, and implementing the NOHP. It helps to produce the Information, Education and Communication (IEC) materials and the Behavior Change Communication (BCC) materials for wider dissemination to states. Its mandate is also to train oral and general health care workers and teachers in imparting oral health in various promotional activities.

India has a large number of dental schools (*n* = 315), which is one of the highest in the world, producing ~25,000 graduates and 5,000 postgraduates in a year [54]. There are more than 400 medical colleges, 640 district hospitals, and 5,335 community health centers with a dental department. In addition, private dental practitioners and corporate hospitals provide dental services. Each of the dental schools and district hospitals has a mobile dental van for community outreach service. Some non-governmental organizations like HelpAge India, Voluntary Health Organization of India and Indian Dental Association undertake various oral health promotion activities and Tobacco Intervention Initiative (TII). However, all of the above listed facilities are designed to serve the population in general and there is no special geriatric oral health care program. Occasionally, some district health cells and dental schools provide “free denture” services for older adults.

There are a number of challenges for geriatric oral health care in India. First, as healthcare is a state-controlled system, planning and implementation of healthcare schemes differ in different states. At present, India has no independent oral health policy. Second, oral healthcare has the lowest share in the overall health budget. Health insurance which is offered to only 10% of the people in the organized sector usually only covers emergency dental treatments. Third, awareness about oral health prevention and various government schemes is poor due to the low literacy level among the older adults and hence their poor utilization. Fourth, the dentist to population ratio is very low in the rural areas where over 71 % of the older adults reside. Fifth, at the undergraduate level, there is very little training in geriatric care embedded in the dental disciplines, with the result that dental graduates are not oriented toward geriatric care. At the postgraduate level, there are no geriatric dentistry courses.

Recommendations to strengthen geriatric oral health care in India have been proposed [55]. These include a shift from individual treatment-based to community-based health promotion and prevention, better utilization of the para-dental workforce for oral health education and basic oral health care, provision of incentives to newly graduated dentists to set up dental clinics in rural areas, effective use of mobile dental clinics to serve the older adults in remote areas, provision of regular oral health care services to the homebound older adults and those in old-age homes, and offering post-graduate diploma and degree programs in geriatric dentistry.

### Japan

Since 2005, the aging rate (aged 65 years and over) of the Japanese population has been the highest in the world. It took only 24 years from the aging rate of 7% (aging society) in 1970 to become 14% (aged society), and this was much faster than in Germany (40 years) and in the United States (72 years). Only 13 years after that, Japan's aging rate reached over 21% (super-aged society). Therefore, Japan experienced an extremely rapid population aging in 40 years, which no other country had ever experienced. In 2020, Japan's older adult population was estimated to be over 36 million, an aging rate of 28.7%. Additionally, it is predicted that the so-called baby boomer generation born after World War II (1947–49) will reach the age of 75 by 2025, which means that a quarter of the Japanese population will be over 75 years old. Among the various social problems that accompany aging (the so-called “2025 problems”), the most serious ones are medical care, long-term care, and welfare.

As a result of these social problems, various efforts have been made in dentistry over the last 10 years with the collaboration of the Japanese government, the Japan Dental Association and academic societies in the field of dentistry. In Japan, most of the medical care and long-term care services are covered by public health insurance systems [56]. While it is an environment in which an efficient healthcare system can be easily designed, most of the medical and long-term care professionals who implement it are in private practice. Therefore, it is difficult to change the old methods drastically. In dentistry, at the beginning of this century it was necessary to switch from the conventional “main complaint response type” to the “healthcare type” as the ideal approach for providing clinical dental services. It has also emphasized the need to focus not only on the control of dental caries and periodontal diseases in children and adults, but also on the prevention and control of oral function deterioration in older adults. According to the national oral health surveys conducted by the Japanese government every 6 years, dental health seems to be improving year by year [25] (Table 1). However, it should be noted that the number of older adults requiring long-term care is increasing as the average life expectancy increases, and the challenges in oral health management are increasing.

Most of the dental treatment in Japan has been provided in dental offices, but the system has been re-designed to increase the opportunities to accommodate older adults living at home or in long-term care facilities, and older patients in hospital. Examples of improving access include the addition of fees for home-visits in medical insurance, the inclusion of fee for oral care and oral functional rehabilitation, as well as reimbursement for the cost of oral care for maintaining oral function in long-term care insurance. Maintaining the oral function of older adults is important not only for maintaining quality of life and reducing the burden of long-term care, but also for preventing aspiration pneumonia and reducing the burden of medical care. The cost of maintaining oral function has been part of the long-term care insurance since 2008, and dentists and dental hygienists have worked in collaboration with physicians and long-term care professionals since 2012. However, in reality, this system is used by only a few long-term care facilities [57]. One of the reasons is that the number of dental professionals who wish to work in the long-term facilities is small, due to the working conditions and their lack of knowledge and skills.

On the other hand, in the medical insurance system, the cost of new tests for oral function such as masticatory ability, occlusal force and tongue pressure have been set in 2018, and the additional cost is allowed when controlling oral hypofunction [58] for older adults by comprehensive diagnosis. Seven sub-symptoms (poor oral hygiene, oral dryness, reduced occlusal force, decreased tongue-lip motor function, decreased tongue pressure, decreased masticatory function, and deterioration of swallowing function) are set as diagnostic items for oral hypofunction. If three or more sub-symptoms are identified positive, they are subject to be managed and reassessed every 6 months. It has been reported that the implementation rate of tests and management for oral hypofunction at 1 year and 2 months after the introduction to medical insurance system was only 1.2% among the 1.88 million first-visit patients aged 65 and over [59]. This indicates that the penetration rate is still very low which might be due to the conservative attitude of private dentists as well as the cost for introducing diagnostic equipment.

In Japan, reforms and continuous reviews of the medical insurance system and the long-term care insurance system are being carried out, and efforts are being made by academic societies and educational institutions to train dental care workers who can be more active in the super aged society. The Japanese Society of Gerodontology (JSG) announced the “Dental Innovation Roadmap to 2040” in 2018, in which the three pillars that support a healthy and long-lived society are oral health management, dentistry in the comprehensive community care system and dental service in terminal care [60]. In order to achieve the goal, JSG states that it is important not only to carry out academic research and human resource development by certifying dentists, but also to work on the medical insurance system and cooperate with regional dental associations.

### Switzerland

There is no Swiss-wide systematic evaluation of oral health, but there have been data from regular Swiss health surveys since 1992. The latest data related to oral health stem from the 2012 survey [26]. According to that survey, the rate of edentulism was 6.5% among the group aged 65–74 years, 8.6% for the group aged 75–84 years and 15.1% for the oldest group aged 85 years or more. Apart from this, there are data available from the largest long-term care facility in Zurich with data from 2010 to 2011 [27]. Furthermore, there are data from the dental consultation of the Division of Geriatrics, University of Bern Hospital, from 2012 [28].

The dental care of the Swiss population is based on self-responsibility of the patient and on the knowledge that tooth loss is mostly avoidable. With few exceptions (serious, unavoidable illness: cancer, genetic diseases, etc.), dental care is excluded from the basic health insurance. The list of exceptions is definitive and does not comprise age-related diseases like dementia or hyposalivation. Healthcare is largely organized by Switzerland's individual communes and there are Chief Dental Officers in most of the 26 cantons. This exceptional organization for a relatively small country is the result of historic developments that date several 100 years back. Currently, there are ~4,800 dentists in Switzerland and 85–90% of dental care is paid out-of-pocket; Majority (73%) of the population sees a dentist at least once a year, predominantly for regular check-ups [61].

As there is no country-wide responsibility for oral health care by the Swiss government, the Swiss Dental Association (SSO) and the Swiss Society for Gerodontology and Special Care Dentistry (SSGS) are the main driving forces to advise on oral care for the older adults. Furthermore, there are several non-connected initiatives within the 26 cantons that often aim to implement the recommendations of the SSO and SSGS [62]. These recommendations comprise: (1) systematic and repeated training of the nursing staff in oral and dental care, in practice and theory, by dental specialists or experienced nurses; (2) a standardized dental examination by a dentist when entering the nursing home; (3) regular implementation of effective oral/dental and prosthesis care measures carried out regularly in accordance with the above recommendations and the documentation for each resident by the nursing staff; (4) regular professional oral hygiene by prophylaxis assistants or by dental hygienists; and (5) cooperation with a consultant dentist for advice and support.

There are non-binding recommendations for older adults who are dependent on care, but the effectiveness of these measures has not been evaluated so far. The main obstacle for implementing a universal health care program for senior citizens in Switzerland is the exceptional and independent organization between the cantons and that oral care is excluded from the basic health care insurance. There are current and past political initiatives to include oral care in the health care system for persons who are dependent on care, as they cannot be made responsible for their own oral hygiene. Furthermore, a nation-wide systematic assessment of the oral health status is needed to better understand the specific requirements of the older Swiss population in regard to oral care.

### Thailand

The population of older adults (aged 60 or older) in Thailand was about 20% of the total population in 2020 and the number is projected to grow rapidly [1]. Most of the older adults live at home in the community. However, 21.4% of them need community-based LTC services [63]. The 8th Thai national oral health survey reported that in 2017, “the young-old” (aged 60–74) had on average 18.6 natural teeth while the old-old (aged 80–85) had 9.9 natural teeth [29]. About 40% of the young-old and only 11% of the old-old had at least 20 natural teeth and 4 occluding pairs of posterior teeth (in that survey, a pair of molars was counted as one occluding unit). Moreover, about half of both groups had at least one tooth with untreated caries. The prevalence of periodontitis among the young-old was 36.3 and 12.2% had 6+mm periodontal pocket. In addition, 8.7% of the young-old and 31% of the old-old were completely edentulous. Finally, 38.6% of the young-old group had at least one dental visit in the past year.

With supportive policies for public health infrastructure such as the National Health Development Strategic Plan for the Elderly (2013–2023), the Universal Health Coverage (UHC), and the Primary Care Act (2019), the National Oral Health Plan for the Elders 2016–2022 (NOHPE) has become Thailand's main driver for a wide range of programs and activities regarding oral health care for the older adult populations [64]. The NOHPE comprises of four strategic areas: (1) service delivery, (2) research and innovation, (3) workforce and curriculum development, and (4) monitoring and evaluation [64].

The main national policies and implementation include an integration of oral health services with the non-communicable disease (NCD) clinics in community hospitals as well as having a public health dentist and a dental nurse working as part of a sub-district level primary care team nationwide. The services include clinical examination as well as oral cancer screening at least once a year; consultation and referral system for comprehensive dental treatments including a home visit service; training and networking of village health volunteers and members of the community-based senior clubs for daily oral care of both independent and dependent older adults. Most of the essential oral health care procedures are covered under the UHC, especially preventive care as well as basic curative care, including acrylic dentures [64, 65].

As for the educational strategies of the NOHPE [64], most dental schools and community colleges have incorporated content regarding oral health care for older adults into their undergraduate dentistry and dental hygiene curricula. To date, seven governmental dental schools offer a master's degree in gerodontology with financial support from the NOHPE, including full tuition-fee scholarship awarded to five graduate students of each program since 2018. In addition, more than a 100 scholarships for a 4-month certificate course as part of continuing professional development, run by government dentistry and dental hygiene schools have also been available each year since 2017.

In summary, public health infrastructure in Thailand has facilitated the development of oral health care programs for older adults. Nonetheless, securing tax-based budget for the initiatives and sustaining the UHC system is challenging. The recent Primary Care Act and its movement has been promoted as a solution for a resilient and sustainable system [66]. Therefore, dental professionals should strive to be more integrated in primary care as well as to show the continuity of LTC spectrum. However, one of the challenges is to enable dentists and dental nurses to provide and coordinate community oriented and “continuing” person-centered care, rather than traditional chief-complaint-based or acute-care management. In addition, a national level consensus on the competencies of primary care dentists and dental hygienists working in the aged society has not been formulated. Furthermore, only dental personnel working for the Ministry of Public Health hospitals are eligible for educational scholarships. Hence, there are needs to clarify national competency-based curricula and to seek more involvement from other governmental agencies as well as private practitioners.

### The United Kingdom

Epidemiological data on the oral health status of older adults in the United Kingdom (UK) is collected by the Adult Dental Health Survey (ADHS) [30]. The first national ADHS was conducted in 1968 and has been repeated almost every decade since, with the most recent ADHS conducted in 2009 and a further survey planned for 2021. Significant improvements in the oral health of older adults within the UK have been recorded in consecutive surveys, particularly in relation to natural tooth retention: 6% of the UK population were edentulous in 2009 compared to 37% in 1968 [30, 67]. However, chronic dental diseases including caries have become more prevalent during this period: 27% of the adults aged 65–74 years had dental caries in 2009 whilst this figure increased to 40% for those aged 75–84 years [67]. The 2009 Survey reported that 73% of all adults had exposed root surfaces and this increased to 90% for those aged over 55 years. The same survey reported that 11% of the 55–64 years old adults had active root caries compared with 20% of those aged 75–84 years [30, 68].

Oral healthcare services for older adults in the UK is provided through the publicly funded National Health Service (NHS) with the vast majority of care offered through independent general dental practices within primary care. The majority of older patients pay a proportion of treatment charges but some are exempted based on financial income levels. A broad range of specified dental treatments are offered, including direct and indirect restorations, and prosthodontic and periodontal care although there is limited access to dental implant treatment. Although the organization of dental services varies throughout the constituent parts of the UK, all systems reward dentists based on levels of operative clinical activity with less emphasis placed on prevention. Currently in the UK, geriatric dentistry is not recognized as a clinical specialty and there are currently no formal taught postgraduate courses offered in this area [69]. Since 2005, special care dentistry has been recognized as a clinical specialty and management of dependent older adults within residential nursing homes falls within the remit of these clinicians and those in the salaried Community Dental Service.

Older dependent adults within residential care are worth particular consideration when discussing issues around oral health provision within the UK. Strategies for this population are to prevent oral diseases and to reduce pain and co-morbidity as summarized in a recent National Institute for Health and Care Excellence (NICE) guideline which aims to maintain and improve the oral health of care home residents [70]. Unfortunately, a recent investigation by the Care Quality Commission (CQC) in England showed the current provision and service to be very poor with limited knowledge and update of the NICE guideline within homes [71]. Current research is ongoing into better implementation of the NICE guideline within residential care as part of the Improving the Oral Health of Older People in Care Homes (TOPIC) study [72].

### The United States

The current population of the United States (US) is ~332 million persons, of which 52.4 million are aged 65 and older and make up 15.8% of the entire US population [73]. The aging of this older population is driven by the “Baby Boom” generation (born between 1945 and 1964), and for the next 10 years, each day 10,000 of them will turn 65 [74]. The majority of the US aging population is dentate. However, there are disparities in the prevalence of edentulousness due to variance in socio-economic and educational status. In 2018, the overall rate of edentulousness was 12.9% for persons aged 65 and older [31]. Edentulousness increases with age and was about 40% among low-income older adults with less education, which is double that of older adults with higher-incomes [31, 75]. The most recent data on the prevalence of dental caries among older adults in the US showed that 96% had had caries. The prevalence of untreated caries was 16%, and the mean DMFT score was 16.7 [32]. The percentage of older adults presenting with one site of clinical attachment loss (loss of periodontal tissue attachment to the root of tooth) of 3 mm or more was found to be 96.4%, whereas 62.3% had a clinical attachment loss of 5 mm or more [33].

In the US, dental health is not considered to be a right but rather a privilege, and is a reflection of its culture, which emphasizes independence and individualism. Although there is a federally supported National Institute of Dental and Craniofacial Research and there are federally qualified health centers with dental services, each state is responsible for its oral healthcare policies. The majority of states have a dental director. Funding for dental care is primarily out-of-pocket, as only 29.2% of older adults have dental health insurance and this percentage declines with age [76].

In 1965, there was a major change in social legislation, which included Medicare and Medicaid. Medicare was a federally funded medical health insurance for older adults. However, this program did not include dental care, except certain oral surgery procedures and non-dental oral soft tissue lesions [77]. Medicaid was a joint federal and state program, which varied by states. In all states, Medicaid provides healthcare coverage for some low-income people, families and children, pregnant women, older adults, and people with disabilities. In some states this program covers all low-income adults below a certain income level (on average it is 138% of the federal poverty level). However, adult dental care was optional and depended on individual state funding. Less than half of the states currently provide comprehensive dental care. As a result, there is enormous variation on what dental procedures states will allow and the level of reimbursement for specific procedures. In addition, many dentists in private practice do not accept Medicaid due to inadequate reimbursement and the administrative overhead costs required to receive reimbursement [78, 79].

In 2010, the Affordable Care Act (ACA) was passed by Congress and provided a number of rights and protections to make healthcare more accessible and affordable. Ten categories of service were required under ACA, such as doctors' services, inpatient and outpatient hospital care, prescription drug coverage, pregnancy and childbirth, and mental health services. However, the ACA does not require insurance plans to include dental care. In fact, if dental insurance is purchased as a stand-alone policy, it will not receive federal subsidy tax credits under the law. ACA also allowed states to expand their Medicaid programs to cover more people with low incomes, and these programs could include reimbursement for dental care [79].

Many US older adults are at risk for poor oral health, which can have a negative impact on their systemic and mental health [80]. Older adults with lower socio-economic status have multiple barriers accessing oral healthcare, which includes transportation, finding a dentist educated in geriatric dentistry who is prepared to care for them, paying for dental care, and a lack of understanding about the availability of financial assistance programs [80]. The solution to some of these economic barriers may be the inclusion of dental benefits in the Medicare and the ACA expansion programs. In addition, dental school curricula must increase the amount of time devoted to clinically caring for frail and functionally dependent older adults. Currently, there are only five post-graduate training programs in geriatric dentistry, which is insufficient to provide an adequate number of teachers and mentors to educate the future general dental workforce to care for older adults [81]. Table 2 presents the summary of the oral health policies for older adults in each country/region.

**Table 2:** Summary of current oral health policies for older adult populations in the selected countries/regions.

**Country/region**	**Current oral health policies and schemes**	**Formal training of geriatric dentistry**	**Number of trained dental professionals in geriatric dentistry**	**Geriatric dentistry recognized as a specialty**	**Main challenges or** **future plans**
Australia	Limited large-scale programs addressing the oral health of older adults Older adults with low income (eligible for government pension) are eligible for public dental care but often with a co-payment	No	A small number	No	Shortage of dental personnel, lack of expertise in geriatric dentistry A promising approach is to place oral health therapists in residential aged care facilities to support dental hygiene and referral
Brazil	Older adults are prioritized to have preferential access to oral health care within the primary health care Oral health promotion and prevention, dental clinical care and oral rehabilitation with partial and complete dentures as well as advanced dental treatments are provided	Yes	275	Yes	Limited access to dental services An inefficient bureaucracy
China	Cost of dental treatments is partially covered by the medical insurance system “Free denture” programs for low-income older adults	Yes	Unknown	No	Poor oral health knowledge, economic barriers, perceived lower priority for oral healthcare by older adults Limited dental care resources National Health Commission issued “Healthy Oral Action Plan (2019–2025),” advocating older adults to pay attention to their oral health
Hong Kong	No specific oral healthcare policy for older adults Two public funded dental care service programs for older adults	No	Unknown	No	Shortage of dental personnel, lack of expertise in geriatric dentistry Insufficient collaboration between dental and other primary healthcare workers, and inadequate oral health care training for healthcare providers
India	No specific oral healthcare policy for older adults Occasionally, some district health cells and dental schools provide “free denture” services for older adults	No	Unknown	No	Planning and implementation of healthcare schemes differ in different states Lack of budget Low dentist to population ratio in the rural areas Poor awareness about oral health and government schemes
Japan	Re-designed system to deliver dental care to institutionalized older adults Collaborations among dentists, dental hygienists, physicians and long-term care professionals Costs for testing oral function and controlling oral hypofunction are covered in medical insurance	Yes	Unknown	Yes	Reforms and continuous reviews of the medical insurance system and the long-term care insurance system are being carried out Efforts are being made by academic societies and educational institutions to train dental care workers to be active in the super aged society
Switzerland	Dental care is excluded from the basic health insurance No specific oral healthcare policy for older adults	Yes	Unknown	No	Main obstacle is the exceptional and independent organization between the cantons and that oral care is excluded from the basic health care insurance
Thailand	National Oral Health Plan for the Elders 2016–2022 (NOHPE) is the main driver for a wide range of programs and activities regarding oral health care for older adults	Yes	Unknown	Yes	Lack of budget Challenging for dentists and dental nurses to provide and coordinate community oriented and “continuing” person-centered care, rather than traditional chief-complaint-based or acute care National level consensus on the competencies of primary care dentists and dental hygienists working in the aged society has not been formulated
Australia UK	Oral healthcare services for older adults are provided through the publicly funded National Health Service (NHS) A broad range of dental treatments are offered, including restorative and periodontal care, but there is limited access to dental implant treatment	No	Unknown	No	Better implementation of the National Institute for Health and Care Excellence (NICE) guideline on maintaining and improving the oral health of care home residents
USA	Dental care is primarily out-of-pocket, but only 29.2% of older adults have dental health insurance and this percentage declines with age Medicare, a federally funded medical health insurance for older adults, only covers certain oral surgery procedures and non-dental oral soft tissue lesions Medicaid provides healthcare coverage for low-income people but dental care for older adults is optional and depends on individual state funding	Yes	Unknown	Yes	Older adults with lower socio-economic status have multiple barriers accessing oral healthcare, including transportation, finding a dentist educated in geriatric dentistry, paying for dental care, and a lack of understanding about the availability of financial assistance programs

## Discussion

With the accelerating global aging, we will confront enduring oral health challenges as well as opportunities over the next decades. Countries are adapting to the aging transformation of the society at their own pace. The delivery systems of oral healthcare services vary among different countries and regions, where tailor-made strategies and policies are adopted or reformed to assist achieving better oral health. In some countries, although no specific oral health policies and oral healthcare program/schemes are currently available for the older adult population, these countries are considering developing policy initiatives, long-term plans and innovative approaches on oral healthcare for the aging populations in the near future.

The FDI World Dental Federation, in its report “Vision 2030—Delivering Optimal Oral health for All,” calls for actions to attain empowering, evidence-based, integrated, and comprehensive oral healthcare by 2030 [82]. This forward-looking report proposes three pillar strategies, i.e., universal coverage for oral health, integration of oral and general healthcare, and building a resilient oral health workforce for sustainable development [82]. As few population-oriented oral health promotion activities specially focusing on older adults are available worldwide [83], the strategies proposed by the FDI can be used to guide policymakers to make future policies and plans to improve oral health of older adults. Recently, in line with the FDI strategies, the World Health Organization (WHO) has just published a report on oral health as part of its documents for the 148th session of the Executive Board to reinforce the importance of including oral health within the UHC agenda and the incorporation of oral health into primary health care delivery systems [84]. Although this approach is designed for the whole population, the strategies are also applicable for older adults. The WHO recommends countries to establish oral health programs to meet the needs of the older adults, and to integrate oral health into national health policies which would help to improve the oral health status and quality of life of this population group [83]. In addition, it is recommended to implement oral health promotion and disease prevention programs based on the common risk factors approach [85].

UHC, aiming to reduce inequities regarding access to healthcare, has been adopted, reshaped or initiated in a number of countries. At present, oral healthcare is not included or is limited in the UHC in many countries. Even when incorporated into UHC, oral healthcare is not a prominent part of the program, since not all necessary oral health services are covered. For instance, the expenditure for prosthodontic treatment is not fully covered by the UHC in China. Subsidized oral healthcare services for older adults vary from country to country depending on the oral health situations of the people and the level of economic development of the country.

Oral health is an essential part of overall health, and oral diseases commonly share risk factors with NCDs. Integration of oral healthcare into general healthcare can lead to more effective prevention and management of oral diseases, resulting in improved overall health and well-being [82]. This strategy has been adopted in some countries such as Japan and Thailand, and it is expected to be gradually accepted by more countries in the near future. Dental professionals acting alone are not sufficient to deliver sustainable, health-needs-based, and people-centered healthcare. Intensive collaborations with a wide range of healthcare workers are strongly advocated [82].

Different workforce models have been employed in different places where oral health providers at various levels, such as dental therapists, hygienists, and even trained nursing care providers, can help to improve older adults' access to oral health services. Currently, many countries still rely on a dentist-centered mode to provide oral health services. The primary oral healthcare and prevention services are usually inadequate to cope with the huge need for oral healthcare among the large number of older adults. The shift to evidence-based people-centered healthcare mode rather than traditional chief-complaint-based management mode led by dentists is happening in several countries around the world.

Dental education is the foundation for establishment of a proper oral healthcare workforce. Considering the global aging trend, reform of the current dental education curricula is needed to produce responsive and competent dental professionals to serve the growing number of older adults. Although geriatric dentistry has been incorporated into dental education curricula and recognized as a clinical specialty in some countries, having formal postgraduate course/programs or training in geriatric dentistry is uncommon around the world. The number of specialists in geriatric dentistry is grossly insufficient to meet the growing demands of the aging society. Besides, there is shortage of educators and mentors in geriatric dentistry to teach and train the future dental workforce to care for older adults. It should be pointed out that graduation from dental school is never the end of learning and training, since the study period in dental schools is quite limited and technical innovations continue to develop. Life-long learning and continuing education programs are important for dental professionals, which empower them to adapt to the transformation of the aging society and to provide optimal oral health services for the aging population.

As a preliminary exploratory study, there are a number of limitations in the present study. Rather than conducting a review covering a full scale of the world's situations, we purposely selected nine representative countries from various continents to present a picture of the current status. We may not be able to generate a comprehensive analysis report, but we tried to present a general overview of the global situation. These selected countries include the most populous ones (e.g., Brazil, China, India, US) that make up more than half of the world population, and the largest economic entities (China, Japan, UK, US). We covered countries in various continents, including Asia (China including Hong Kong, India, Japan and Thailand), Australia, Europe (Switzerland, UK), North America (US), and South America (Brazil). The only geographical region (continent) that was not covered in this project is Africa, which has the least aging population. In this descriptive study, we did not attempt to conduct policy analysis nor to compare oral health policies and schemes in different countries regarding financing and reimbursement, thereby future studies are needed.

## Conclusion

Individual countries are adopting tailor-made strategies to deliver oral health services for the increasing number of older adults in the aging society. Expansion of dental education and incorporation of clinical training in geriatric dentistry are needed to produce responsive and competent dental professionals to meet the demands of aging societies around the world.

## Author Contributions

All authors listed have made a substantial, direct and intellectual contribution to the work, and approved it for publication.

## Conflict of Interest

The authors declare that the research was conducted in the absence of any commercial or financial relationships that could be construed as a potential conflict of interest.

## Publisher's Note

All claims expressed in this article are solely those of the authors and do not necessarily represent those of their affiliated organizations, or those of the publisher, the editors and the reviewers. Any product that may be evaluated in this article, or claim that may be made by its manufacturer, is not guaranteed or endorsed by the publisher.

## References

[1] United Nations Department of Economic and Social Affairs Population Division. World Population Ageing. (2019). Available online at: https://www.un.org/development/desa/pd/sites/www.un.org.development.desa.pd/files/files/documents/2020/Jan/un_2019_worldpopulationageing_report.pdf

[2] Glick M Williams DM Kleinman DV Vujicic M Watt RG Weyant RJ. A new definition for oral health developed by the FDI World Dental Federation opens the door to a universal definition of oral health. Br Dent J. (2016) 221:792–3. 10.1038/sj.bdj.2016.953 27981999

[3] Dorfer C Benz C Aida J Campard G. The relationship of oral health with general health and NCDs: a brief review. Int Dent J. (2017) 67(Suppl. 2):14–8. 10.1111/idj.12360 29023744 PMC9378917

[4] Wilson W Taubert Kathryn A Gewitz M Lockhart Peter B Baddour Larry M Levison M. Prevention of infective endocarditis. Circulation. (2007) 116:1736–54. 10.1161/CIRCULATIONAHA.106.183095 17446442

[5] Chapple IL Genco R Working Group 2 of the Joint EFPAAPw. Diabetes and periodontal diseases: consensus report of the Joint EFP/AAP Workshop on Periodontitis and Systemic Diseases. J Periodontol. (2013) 84(4 Suppl.):S106–12. 10.1902/jop.2013.1340011 23631572

[6] Kassebaum NJ Smith AGC Bernabé E Fleming TD Reynolds AE Vos T. Global, regional, and national prevalence, incidence, and disability-adjusted life years for oral conditions for 195 countries, 1990-2015: a systematic analysis for the global burden of diseases, injuries, and risk factors. J Dent Res. (2017) 96:380–7. 10.1177/0022034517693566 28792274 PMC5912207

[7] Kassebaum NJ Bernabe E Dahiya M Bhandari B Murray CJ Marcenes W. Global burden of untreated caries: a systematic review and metaregression. J Dent Res. (2015) 94:650–8. 10.1177/0022034515573272 25740856

[8] Kassebaum NJ Bernabé E Dahiya M Bhandari B Murray CJL Marcenes W. Global burden of severe periodontitis in 1990-2010: a systematic review and meta-regression. J Dent Res. (2014) 93:1045–53. 10.1177/0022034514552491 25261053 PMC4293771

[9] Kassebaum NJ Bernabe E Dahiya M Bhandari B Murray CJ Marcenes W. Global burden of severe tooth loss: a systematic review and meta-analysis. J Dent Res. (2014) 93(7 Suppl.):20S−8. 10.1177/0022034514537828 24947899 PMC4293725

[10] GBD 2019 Diseases and Injuries Collaborators. Global burden of 369 diseases and injuries in 204 countries and territories, 1990-2019: a systematic analysis for the Global Burden of Disease Study 2019. Lancet. (2020) 396:1204–22. 10.1016/S0140-6736(20)30925-9 33069326 PMC7567026

[11] GBD 2016 Healthcare Access and Quality Collaborators. Measuring performance on the Healthcare Access and Quality Index for 195 countries and territories and selected subnational locations: a systematic analysis from the Global Burden of Disease Study 2016. Lancet. (2018) 391:2236–71. 10.1016/S0140-6736(18)30994-2 29893224 PMC5986687

[12] Satur J. Oral health and dental services. In: Willis E, Reynolds L Rudge T editors. Understanding the Australian Health Care System. 4th ed. Chatswood, NSW: Elsevier Australia (2020).

[13] Oral Health Monitoring Group. Healthy Mouths, Healthy Lives: Australia's National Oral Health Plan 2015-2024. (2015). Available online at: http://iaha.com.au/wp-content/uploads/2016/02/Australias-National-Oral-Health-Plan-2015-2024_uploaded-170216.pdf

[14] Takehara S Wright FC Naganathan V Hirani V Blyth FM Le Couteur DG. A cross-sectional study of perceived dental treatment needs and oral health status in community-dwelling older Australian men: the Concord Health and Ageing in Men Project. Int Dent J. (2021) 71:224–232. 10.1111/idj.12623 34024332 PMC9275195

[15] Peres MA Lalloo R. Tooth loss, denture wearing and implants: findings from the National Study of Adult Oral Health 2017–18. Aust Dent J. (2020) 65:S23–31. 10.1111/adj.12761 32583591

[16] Do LG Peres KG Ha DH Roberts-Thomson KF. Oral epidemiological examination – protocol: the National Study of Adult Oral Health 2017–18. Aust Dent J. (2020) 65:S18–22. 10.1111/adj.12760 32583582

[17] Kapellas K Roberts-Thomson KF. National Study of Adult Oral Health 2017–18: root caries. Aust Dent J. (2020) 65:S40–6. 10.1111/adj.12763 32583586

[18] Chrisopoulos S Harford J Ellershaw A. Oral Health and Dental Care in Australia: Key Facts and Figure 2015. Canberra, ACT: Australian Institute of Health and Welfare (2016).

[19] AIHW. Oral Health and Dental Care in Australia: Cat. 231. Canberra, ACT: AIHW (2020). Available online at: https://www.aihw.gov.au/reports/den/231/oral-health-and-dental-care-in-australia/contents/summary

[20] Brasil Ministério da Saúde Secretaria de Atenção à Saúde. SB Brasil 2010: Pesquisa Nacional de Saúde Bucal: resultados principais. (2012). Available online at: https://bvsms.saude.gov.br/bvs/publicacoes/pesquisa_nacional_saude_bucal.pdf

[21] People's Medical Publishing House. The Fourth National Oral Health Epidemiological Survey Report. Wang X, editor. Beijing: People's Medical Publishing House (2018).

[22] Department of Health. Oral Health Survey 2011. (2011). Available online at: https://www.toothclub.gov.hk/en/en_pdf/Oral_Health_Survey_2011/Oral_Health_Survey_2011_WCAG_20141112_(EN_Full).pdf

[23] Shah N Mathur VP Kant S Gupta A Kathuria V Haldar P. Prevalence of dental caries and periodontal disease in a rural area of Faridabad District, Haryana, India. Indian J Dent Res. (2017) 28:242–7. 10.4103/ijdr.IJDR_370_16 28721985

[24] Shah N Pandey R Duggal R Mathur V Rajan K editors. Oral Health in India: A Report of the Multi Centric Study, Directorate General of Health Services, Ministry of Health and Family Welfare, Government of India and World Health Organisation Collaborative Program (2007).

[25] Ministry of Health Labour and Welfare. Overview of the Survey of Dental Diseases. (2016). Available online at: https://www.mhlw.go.jp/toukei/list/dl/62-28-02.pdf

[26] Schneider C Zemp E Zitzmann NU. Oral health improvements in Switzerland over 20 years. Eur J Oral Sci. (2017) 125:55–62. 10.1111/eos.12327 28045197

[27] Brändli-Holzer B. Orale Gesundheit und Mundhygiene von neueingetretenen Bewohnern eines Pflegezentrums der Stadt Zürich. (2012). Available online at: http://www.generation-sans-caries.ch/fileadmin/media/pdf/diss_braendli_2012.pdf

[28] Katsoulis J Schimmel M Avrampou M Stuck AE Mericske-Stern R. Oral and general health status in patients treated in a dental consultation clinic of a geriatric ward in Bern, Switzerland. Gerodontology. (2012) 29:e602–10. 10.1111/j.1741-2358.2011.00529.x 21726274

[29] Bureau of Dental Health. The 8th National Oral Health Survey, Thailand 2017. (2018). Available online at: http://dental2.anamai.moph.go.th/ewt_dl_link.php?nid=2423&filename=dental_health_survey

[30] Official Statistics National Statistics Survey. Adult Dental Health Survey 2009 - Summary Report and Thematic Series. (2011). Available online at: https://digital.nhs.uk/data-and-information/publications/statistical/adult-dental-health-survey/adult-dental-health-survey-2009-summary-report-and-thematic-series

[31] Fleming E Afful J Griffin SO. Prevalence of tooth loss among older adults: United States, 2015-2018. NCHS Data Brief. (2020) 1–8. 32600520

[32] Centers for Disease Control and Prevention. Oral Health Surveillance Report, 2019. Trends in Dental Caries and Sealants, Tooth Retention, and Edentulism, United States 1999-2004 to 2011-2016. Available online at: https://www.cdc.gov/oralhealth/publications/OHSR-2019-index.html

[33] Eke PI Thornton-Evans GO Wei L Borgnakke WS Dye BA Genco RJ. Periodontitis in US adults: National Health and Nutrition Examination Survey 2009-2014. J Am Dent Assoc. (2018) 149:576–88.e6. 10.1016/j.adaj.2018.04.023 29957185 PMC8094373

[34] Allin S Farmer J Quiñonez C Peckham A Marchildon G Panteli D. Do health systems cover the mouth? Comparing dental care coverage for older adults in eight jurisdictions. Health Policy. (2020) 124:998–1007. 10.1016/j.healthpol.2020.06.015 32712013

[35] COAG Health Council. Healthy Mouths, Healthy Lives: Australia's National Oral Health Plan 2015–2024. Australian Government Adelaide, SA (2015). Available online at: https://www.coaghealthcouncil.gov.au/Publications/Reports/ArtMID/514/ArticleID/81/Australias-National-Oral-Health-Plan-2015-2024

[36] Slack-Smith LM Hearn L Wilson DF Wright F. Geriatric dentistry, teaching and future directions. Aust Dent J. (2015) 60(Suppl. 1):125–30. 10.1111/adj.12291 25762049

[37] Hearn L Slack-Smith L. Oral health care in residential aged care services: barriers to engaging health-care providers. Aust J Prim Health. (2015) 21:148–56. 10.1071/PY14029 25155109

[38] Chen R Irving M Clive Wright FA Cunich M. An evaluation of health workforce models addressing oral health in residential aged care facilities: a systematic review of the literature. Gerodontology. (2020) 37:222–32. 10.1111/ger.12475 32478960

[39] Wallace J Blinkhorn A Blinkhorn F. A qualitative study examining the preparedness of dental hygiene students for a service-learning placement in residential aged care. Int J Dent Hyg. (2017) 15:30–6. 10.1111/idh.12157 26086397

[40] Wallace JP Mohammadi J Wallace LG Taylor JA. Senior smiles: preliminary results for a new model of oral health care utilizing the dental hygienist in residential aged care facilities. Int J Dent Hyg. (2016) 14:284–8. 10.1111/idh.12187 26608383

[41] Peres MA Barbato PR Reis SCGB Freitas CHSdM Antunes JLF. Perdas dentárias no Brasil: análise da Pesquisa Nacional de Saúde Bucal 2010. Rev Saúde Pública. (2013) 47:78–89. 10.1590/S0034-8910.2013047004226 24626584

[42] Marques RAdA Antunes JLF Sousa MdLR Peres MA Frazão P. Prevalência e extensão da cárie dentária radicular em adultos e idosos brasileiros. Rev Saúde Pública. (2013) 47:59–68. 10.1590/S0034-8910.2013047004365 24626582

[43] Pucca GA Jr Gabriel M de Araujo ME de Almeida FC. Ten years of a national oral health policy in Brazil: innovation, boldness, and numerous challenges. J Dent Res. (2015) 94:1333–7. 10.1177/0022034515599979 26316461

[44] Hebling E Mugayar L Dias PV. Geriatric dentistry: a new specialty in Brazil. Gerodontology. (2007) 24:177–80. 10.1111/j.1741-2358.2007.00155.x 17696896

[45] The Seventh National Census. National Bureau of Statistics. (2021). Available online at: http://www.stats.gov.cn/tjsj/sjjd/202105/t20210512_1817336.html (accessed July 6, 2021).

[46] Jiao J Jing W Si Y Feng X Tai B Hu D. The prevalence and severity of periodontal disease in Mainland China: data from the Fourth National Oral Health Survey (2015-2016). J Clin Periodontol. (2021) 48:168–79. 10.1111/jcpe.13396 33103285

[47] Du M Petersen PE Fan M Bian Z Tai B. Oral health services in PR China as evaluated by dentists and patients. Int Dent J. (2011) 50:250–6. 10.1111/j.1875-595X.2000.tb00561.x 15988882

[48] Ministry of Health of the People's Republic of China. China Health Statistical Yearbook (2011) Beijing: Peking Union Medical College Press (2012). Available online at: http://www.nhc.gov.cn/htmlfiles/zwgkzt/ptjnj/year2011/index2011.html

[49] Liu J Zhang SS Zheng SG Xu T Si Y. Oral health status and oral health care model in China. Chin J Dent Res. (2016) 19:207–15. 10.3290/j.cjdr.a37145 27995225

[50] The State Council. Beijing Deepen Implementation Project on Medical and Health System 2010-2011. (2010). Available online at: http://www.gov.cn/gzdt/2010-06/13/content_1627249.htm

[51] Gao SS Chen KJ Duangthip D Lo ECM Chu CH. Oral health care in Hong Kong. Healthcare. (2018) 6:45. 10.3390/healthcare6020045 29751605 PMC6023485

[52] Yang SX Leung KC Jiang CM Lo EC. Dental care services for older adults in hong kong—a shared funding, administration, and provision mode. Healthcare. (2021) 9:390. 10.3390/healthcare9040390 33915802 PMC8067220

[53] Legislative Council Secretariat. Dental Care Services and Relevant Manpower Planning. (2020). Available online at: https://www.legco.gov.hk/yr19-20/english/panels/hs/papers/hs20200320cb2-711-6-e.pdf

[54] Dental Council of India. Welcome to Dental Council of India. Available online at: https://www.dciindia.gov.in/

[55] Shah N. Geriatric oral health issues in India. Int Dent J. (2001) 51(3 Suppl.):212–8. 10.1002/j.1875-595X.2001.tb00869.x 11561881

[56] Ministry of Health Labour and Welfare. Overview of Medical Service Regime in Japan. Available online at: https://www.mhlw.go.jp/bunya/iryouhoken/iryouhoken01/dl/01_eng.pdf

[57] Ohuchi A. Socioeconomic Effects of the Collaboration Between Oral Health Management and Nutrition Management in Long-Term Care Insurance Facilities. (2018). Available online at: https://kaken.nii.ac.jp/en/file/KAKENHI-PROJECT-15K15772/15K15772seika.pdf

[58] Minakuchi S Tsuga K Ikebe K Ueda T Tamura F Nagao K. Oral hypofunction in the older population: position paper of the Japanese Society of Gerodontology in 2016. Gerodontology. (2018) 35:317–24. 10.1111/ger.12347 29882364

[59] Sato Y Kitagawa N Shichita T Hatanaka Y Uchida Y. State of implementation of examination and management of oral hypofunction newly covered by national health insurance -second report. Jpn J Gerodontol. (2021) 35:230–2.

[60] Minakuchi S. Greeting From the President of Japanese Society of Gerodontology. Available online at: https://www.gerodontology.jp/about/greeting.shtml

[61] Marmy O Gubler M Tackenberg M. Steigerung auf hohem Niveau. Swiss Dent J. (2018) 185:411–5.

[62] CURAVIVA_Schweiz. Zahnmedizinische Betreuung in Pflegeheimen. 1st ed. (2017). Available online at: https://www.curaviva.ch/files/3HY2TM5/zahnmedizinische_betreuung_in_pflegeheimen__publikation__curaviva_schweiz__2015.pdf

[63] Aekplakorn W. Thai National Health Examination Survey, NHES V. (2014). Available online at: http://www.thaiheart.org/images/column_1387023976/NHES5_EGATMeeting13Dec13.pdf

[64] Bureau of Dental Health. Report of the National Oral Health Plan for the Elders 2016-2022: Phase 1 (2016-2019). Bangkok: Samcharoenpanich (2020).

[65] Bureau of Dental Health. Organization Structure and Roles of Division of Oral Health Development for Working and Older People. (2016). Available online at: http://dental2.anamai.moph.go.th/ewt_news.php?nid=1924

[66] Sumriddetchkajorn K Shimazaki K Ono T Kusaba T Sato K Kobayashi N. Universal health coverage and primary care, Thailand. Bull World Health Organ. (2019) 97:415–22. 10.2471/BLT.18.223693 31210679 PMC6560367

[67] Steele JG Treasure ET O'Sullivan I Morris J Murray JJ. Adult Dental Health Survey 2009: transformations in British oral health 1968-2009. Br Dent J. (2012) 213:523–7. 10.1038/sj.bdj.2012.1067 23175081

[68] White DA Tsakos G Pitts NB Fuller E Douglas GV Murray JJ. Adult Dental Health Survey 2009: common oral health conditions and their impact on the population. Br Dent J. (2012) 213:567–72. 10.1038/sj.bdj.2012.1088 23222333

[69] Kossioni A McKenna G Müller F Schimmel M Vanobbergen J. Higher education in Gerodontology in European Universities. BMC Oral Health. (2017) 17:71. 10.1186/s12903-017-0362-9 28351394 PMC5371193

[70] NICE. Oral Health for Adults in Care Homes. NICE Guideline [NG48]: National Institute for Health and Care Excellence 2016. (2016). Available online at: https://www.nice.org.uk/guidance/ng48

[71] Care Quality Commission. Smiling Matters: Oral Health in Care Homes. (2019). Available online at: https://www.cqc.org.uk/publications/major-report/smiling-matters-oral-health-care-care-homes

[72] Institute of Epidemiology & Health Care UCL. TOPIC - Oral Health in Care Homes. Available online at: https://www.ucl.ac.uk/epidemiology-health-care/research/epidemiology-and-public-health/research/dental-public-health/research/topic-oral-health

[73] Administration for Community Living. 2019 Profile of Older Americans. (2019). Available online at: https://acl.gov/sites/default/files/Aging%20and%20Disability%20in%20America/2019ProfileOlderAmericans508.pdf

[74] Ettinger RL Marchini L. Cohort differences among aging populations: an update. J Am Dent Assoc. (2020) 151:519–26. 10.1016/j.adaj.2020.04.001 32593354

[75] Griffin SO Griffin PM Li CH Bailey WD Brunson D Jones JA. Changes in older adults' oral health and disparities: 1999 to 2004 and 2011 to 2016. J Am Geriatr Soc. (2019) 67:1152–7. 10.1111/jgs.15777 30698819 PMC7962435

[76] Kramarow EA. Dental care among adults aged 65 and over, 2017. NCHS Data Brief. (2019) 1–8. 31163014

[77] Moss ME. Integrating oral health care services within medicare. N C Med J. (2017) 78:402–5. 10.18043/ncm.78.6.402 29203605

[78] Northridge ME Metcalf SS Birenz SS Kunzel C Wang H Schrimshaw EW. The impact of medicaid expansion on oral health equity for older adults: a systems perspective. J Calif Dent Assoc. (2015) 43:369–77. 26457047 PMC4596543

[79] Reynolds JC Sukalski JMC McKernan SC Damiano PC. Member and dentist narratives about a dental programme for the Medicaid expansion population: a content analysis. Commun Dent Oral Epidemiol. (2019) 47:485–93. 10.1111/cdoe.12490 31441086

[80] Marchini L Reynolds JC Caplan DJ Sasser S Russell C. Predictors of having a dentist among older adults in Iowa. Community Dent Oral Epidemiol. (2020) 48:240–7. 10.1111/cdoe.12521 32043281

[81] Ettinger RL Goettsche ZS Qian F. Postdoctoral teaching of geriatric dentistry in U.S. dental schools. J Dent Educ. (2017) 81:1220–6. 10.21815/JDE.017.079 28966187

[82] FDI World Dental Federation. FDI's Vision 2030 Report Aims to Improve Oral Health and Reduce Oral Health Inequalities Over the Next Decade. (2021). Available online at: https://www.fdiworlddental.org/fdis-vision-2030-report-aims-improve-oral-health-and-reduce-oral-health-inequalities-over-next

[83] Petersen PE Kandelman D Arpin S Ogawa H. Global oral health of older people–call for public health action. Community Dent Health. (2010) 27(4 Suppl. 2):257–67. 21313969

[84] World Health Organization. Oral Health-Achieving Better Oral Health as Part of the Universal Health Coverage and Noncommunicable Disease Agendas Towards 2030. (2020). Available online at: https://apps.who.int/gb/ebwha/pdf_files/EB148/B148_8-en.pdf

[85] Petersen PE Yamamoto T. Improving the oral health of older people: the approach of the WHO Global Oral Health Programme. Community Dent Oral Epidemiol. (2005) 33:81–92. 10.1111/j.1600-0528.2004.00219.x 15725170

